# Follow-up results of microendoscopic discectomy compared to day surgery using percutaneous endoscopic lumbar discectomy for the treatment of lumbar disc herniation

**DOI:** 10.1186/s12891-021-04038-6

**Published:** 2021-02-09

**Authors:** Zhaojun Song, Maobo Ran, Juan Luo, Kai Zhang, Yongjie Ye, Jiazhuang Zheng, Zhi Zhang

**Affiliations:** 1 Spine Surgery Department of Suining Central Hospital, Suining, Sichuan People’s Republic of China; 2 Medical Record Department of Suining Central Hospital, Suining, Sichuan People’s Republic of China; 3 Day Surgery Unit of Suining Central Hospital, Suining, Sichuan People’s Republic of China

**Keywords:** Day surgery, Lumbar disc herniation, Percutaneous endoscopic lumbar discectomy, Microendoscopic discectomy, Disc height, Instability

## Abstract

**Background:**

Percutaneous endoscopic lumbar discectomy (PELD) is satisfactory for hospitalized patients with lumbar disc herniation (LDH). Currently, only a few studies have reported about the day surgery patients undergoing PELD.

**Methods:**

A total of 267 patients with LDH underwent PELD during day surgery and were followed up for at least 3 years. Clinical outcomes were assessed using the visual analog scale (VAS) for leg and lower back pain (VAS-B and VAS-L, respectively) and the Oswestry disability index (ODI). The radiological outcomes, such as lumbar lordosis (LL), sacral slope (SS), the disc-height ratio, and disc instability, were recorded and compared. The clinical effects between patients treated by PELD during day surgery and microendoscopic discectomy (MED) for contemporaneous hospitalized 116 patients with LDH were compared.

**Results:**

Patients treated by PELD had lower blood loss and shorter hospital stay (*P* <  0.001) compared to those treated by MED. VAS-L, VAS-B, and ODI decreased significantly after PELD than before the operation and 3 years postoperatively. The postoperative VAS-B in the PELD group was significantly decreased than in the MED group (*P* = 0.001). The complications rate was 9.4% in the PELD group and 12.1% in the MED group (*P* = 0.471). The 1-year postoperative recurrence rate in the PELD group was much higher than that in MED group (*P* = 0.042). The postoperative LL and SS in the PELD group improved significantly compared to the values in the MED group (*P* <  0.001). According to the disc-height ratio at 3-year follow-up, a significant height loss was observed in the MED group than in the PELD group (*P* = 0.014).

**Conclusions:**

Although the 1-year postoperative recurrence rate was relatively high, the day surgery for LDH undergoing PELD had advantages in terms of less blood loss intraoperatively, short hospital stay, efficacy for back pain, and efficiency to maintain lumbar physiological curvature.

## Introduction

Since the 1980s, Kambin began to use endoscopy or arthroscopy for lumbar disc herniation (LDH), and following the improvement of the spinal endoscopic surgery equipment, percutaneous discectomy has developed rapidly [1, 2]. In 1997, Yeung proposed the Yeung endoscopic spine system, consisting of an endoscope with a 2.8-mm operating channel. The endoscopes and instruments are placed through a working sleeve, and the intervertebral disc is removed from the inside to the outside [3]. Clinical studies have shown that Yeung endoscopic spine system has a satisfactory effect in the treatment of LDH [4]. Hoogland et al. proposed that the transforaminal endoscopic surgical system (TESSYS system) directly releases and decompresses the nerve root through the operating channel into the spinal canal [5]. With the improvement and development of the percutaneous endoscopic lumbar discectomy (PELD) technology, high-quality images of spine anatomy help the surgeons to understand the pathological changes in patients with LDH. PELD has many advantages, such as less trauma, fewer complications, and satisfactory results while minimizing postoperative instability [6–8].

Day surgery, also known as ambulatory surgery, same-day surgery, and one-day surgery, has been carried out in developed countries in Europe and USA for more than a decade. Although previous studies have shown satisfactory clinical results of PELD for hospitalized patients with LDH, only a few studies have reported about the day surgery patients undergoing PELD. Thus, the clinical outcomes of day surgery for LDH using PELD and the related complications need further investigation. The present study described the postoperative clinical outcomes of day surgery for LDH patients undergoing PELD.

## Patients and methods

This was a single center retrospective study. All methods were performed in accordance with relevant guidelines and regulations. A total of 306 consecutive patients with L5-S1 LDH underwent PELD in the Day Surgery Unit of Suining Central Hospital using the SPINENDOS spinal full-endoscopic system (SPINENDOS, Munich, Germany) between March 2015 and 2017.

The inclusion criteria were as follows: 1) diagnosis by computed tomography (CT) and magnetic resonance imaging (MRI) of lumbar vertebra disc; 2) neurological examination, including the typical symptom of back pain and radicular pain and positive results from the femoral nerve stretch test or sciatic nerve stretch test; 3) invalidation of conservative treatment for > 6 weeks and indications of operation and the treatment being expected to be effective. The exclusion criteria were as follows: 1) primary lumbar spinal stenosis, lumbar instability, lumbar spine trauma, lumbar spine tumor, infection, or other pathological conditions; 2) lumbar disc herniation accompanied by underlying diseases that cannot be candidates for operation.

We compared the clinical effects between patients treated by PELD in the Day Surgery Unit and contemporary hospitalized patients (116 cases) with LDH treated by microendoscopic discectomy (MED) using the DCZJ-II MED system (Dragon Crown, Shandong, China). The baseline information of the two groups was not different as shown in Table 1.

**Table 1:** Comparison of the baseline information of the two groups

Groups	PELD	MED	t/χ^**2**^ test	***P***-value
**Male/female**	154 / 113	75 / 41	1.638	0.201
**Age (years)**	47.2 ± 13.2	49.0 ± 10.9	- 1.276	0.203
**BMI**	24.1 ± 3.4	23.6 ± 2.6	- 0.372	0.710
**VAS-B**	1.9 ± 1.4	2.1 ± 1.0	- 1.318	0.188
**VAS-L**	6.8 ± 1.0	6.7 ± 0.9	0.397	0.691
**ODI (%)**	63.5 ± 10.2	62.5 ± 7.3	1.039	0.300

### Surgical technique

Preoperative examinations, including blood routine test, blood biochemistry checking, blood electrolytes, coagulation convention, pretransfusion test, blood type, chest X-ray, electrocardiogram, A-P and lateral plain film of lumbar vertebra, excessive extension and flexion posture of lumbar vertebra, CT and MRI of lumbar vertebra, were applied to patients. PELD was performed under general anesthesia via an interlaminar approach. All surgeries were performed by two experienced senior spine surgeons.

**Fig. 1:**
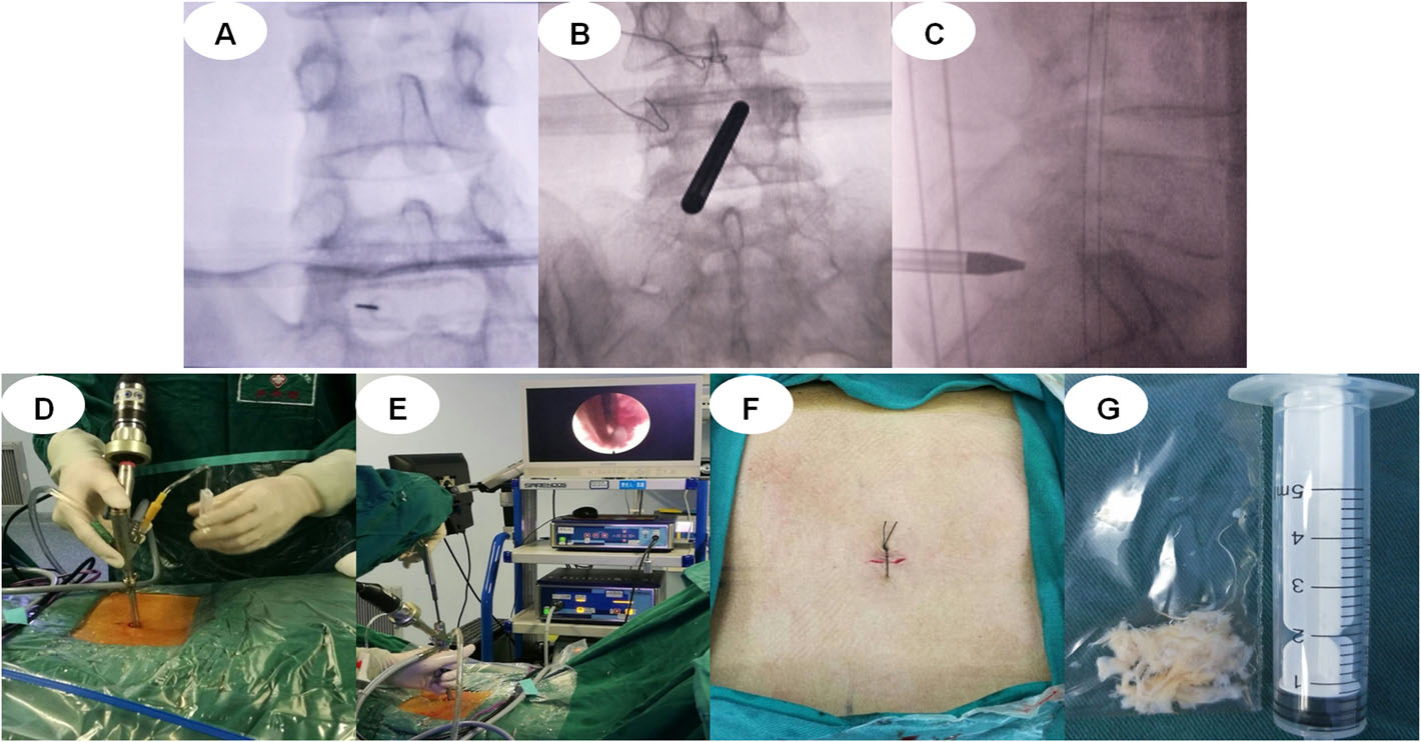
A typical PELD case via interlaminar approach. **a** puncture and radiography; **b** puncture and radiography; **c** puncture and radiography; **d** positioning of the tube; **e** the process of surgical operations; **f** the operative incisions; and **g** the herniated nucleus pulposus is removed. PELD, percutaneous endoscopic lumbar discectomy

**Fig. 2:**
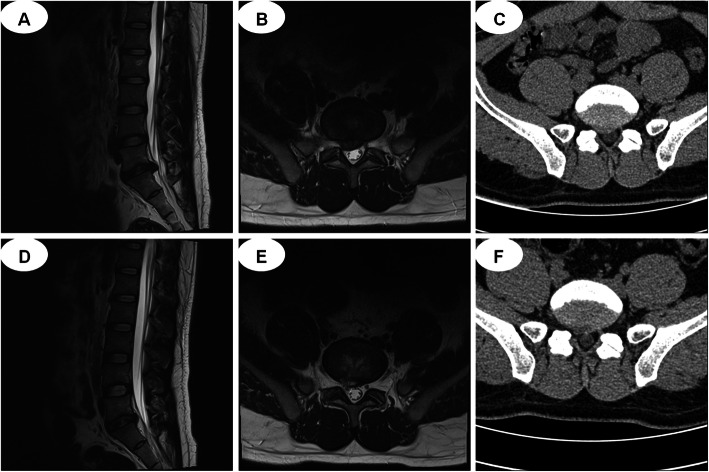
CT and MRI images. **a**-**c** MRI and CT show L5-S1 discal hernia before surgery; **d**-**f** MRI and CT show the herniated nucleus pulposus is removed after surgery. CT, computed tomography; MRI, magnetic resonance imaging

PELD group: The patients were placed in the prone position. Puncture position was guided under the C-arm X-ray perspective machine using the interlaminar approach. 1) In the positive section of the C-arm film, a longitudinal line was drawn along the lumbar spinous process. Another longitudinal line was drawn along the inner edge of the facet that was parallel to the spinous process line. A horizontal line was drawn between the two vertebrae requiring an operation. The point of intersection with the facet edge line is the puncturing point. 2) Interlaminar space was determined by the C-arm. The puncture point was approximately 5 mm away from the midline in the protrusion side of nucleus pulposus of the intervertebral space. 3) A working channel was constructed by making a skin incision approximately 5 mm, extending the guide bar to the ligamentum flavum. 4) The remaining tissues in the work channel were cleaned, and the ligamentum flavum was exposed. The ligamentum flavum, partial vertebral laminae and fat were removed. The working sleeve was rotated into the spinal canal, separated, and the nerve root and nucleus pulposus exposed. The herniated nucleus pulposus was removed, and radiofrequency ablation was performed for annulus fibrosus and nucleus pulposus (Fig. 1). CT and MRI images of lumbar vertebra for the interlaminar approach to the PELD case are shown in Fig. 2.

**Fig. 3:**
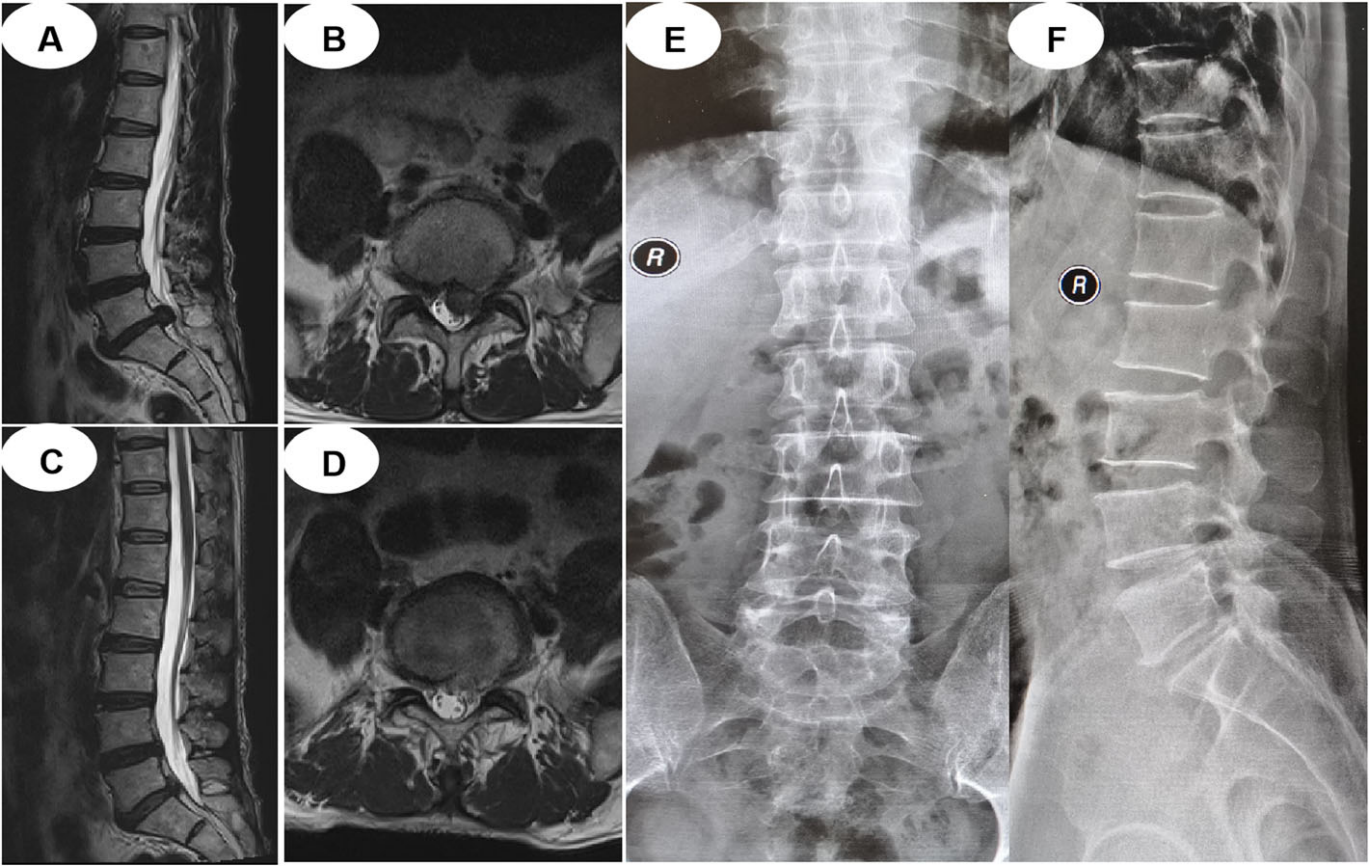
**a** and **b** MRI show L5-S1 discal hernia before MED; **c** and **d** MRI show the herniated nucleus pulposus is removed after surgery; **e** and **f** Postoperative x-ray images

MED group: 1) Surgery was carried out under general anesthesia with the patients in the prone position. 2) A longitudinal skin incision of 20 mm was made approximately 30 mm lateral to the spinous process line. 3) A diameter was inserted towards the inter lamina space after dissection of the fascia under the guidance of C-arm. 4) Dilators were inserted sequentially, and a tubular retractor was placed to the inferior border of the lamina and the medial border of the inferior articular process. 5) Exposing the ligamentum flavum, and removing the adhering soft tissues together with part of the bony structures from the lamina and the articular process. 6) Splitting the ligamentum flavum, and exposing the compressed dural sac and nerve root that were removed carefully to decompress the nerve root. Following discectomy, the intervertebral space was washed with saline solution, and suction drainage was placed. Preoperative MRI, postoperative MRI, and X-ray images are shown in Fig. 3.

### Clinical evaluation

We compared the clinical effects of patients treated with PELD in the Day Surgery Unit and contemporary hospitalized patients (116 cases) with LDH treated by microendoscopic discectomy (MED). Operation time, operative blood loss, hospital stay, and postoperative complications were recorded and analyzed. Clinical follow-ups were conducted each year after the operation by telephone, WeChat (Tencent International Service Pvt. Ltd., Shenzhen, China), and a post-surgery questionnaire.

**Fig. 4:**
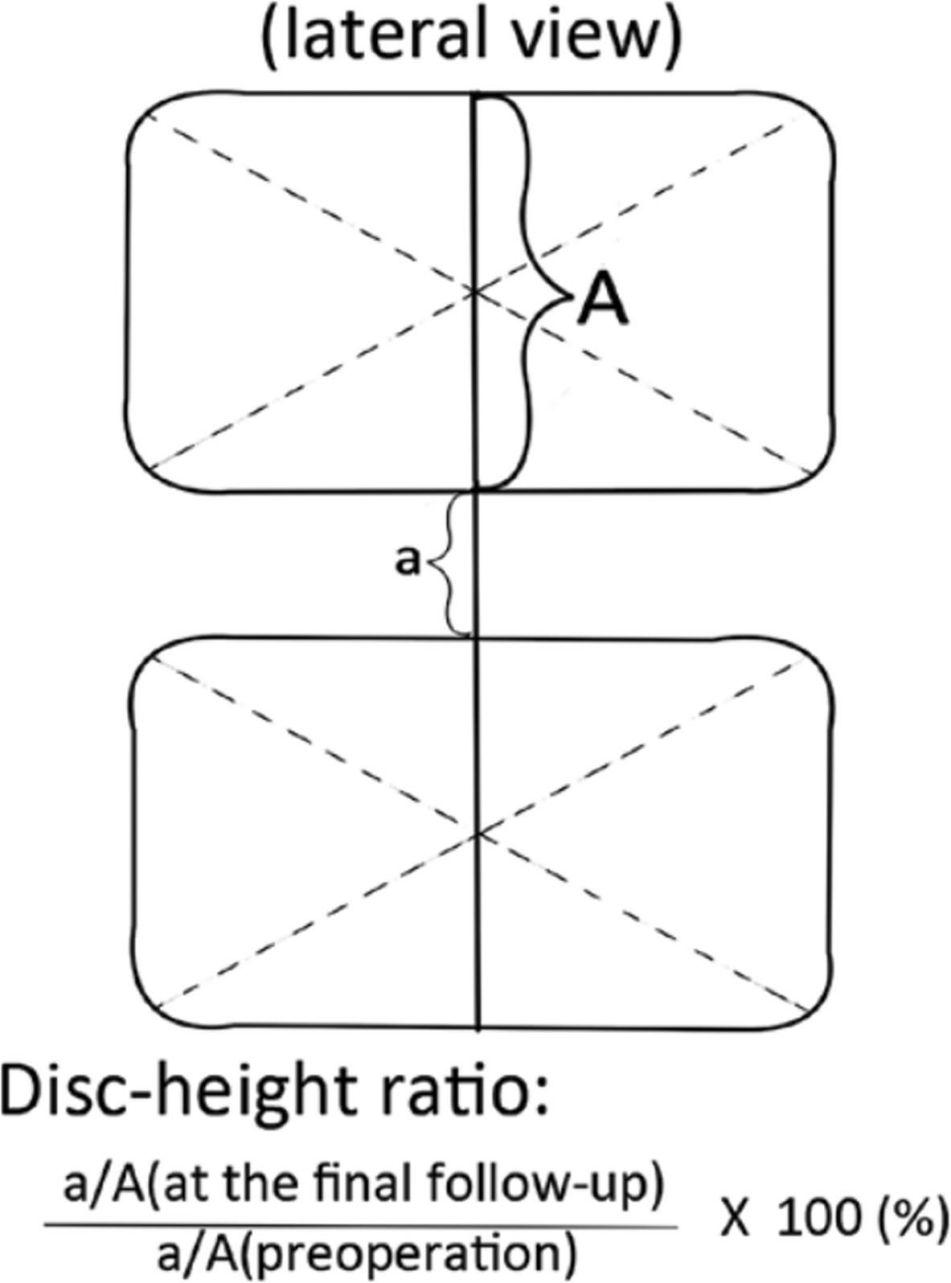
Mochida method for measuring the disc height ratio

**Fig. 5:**
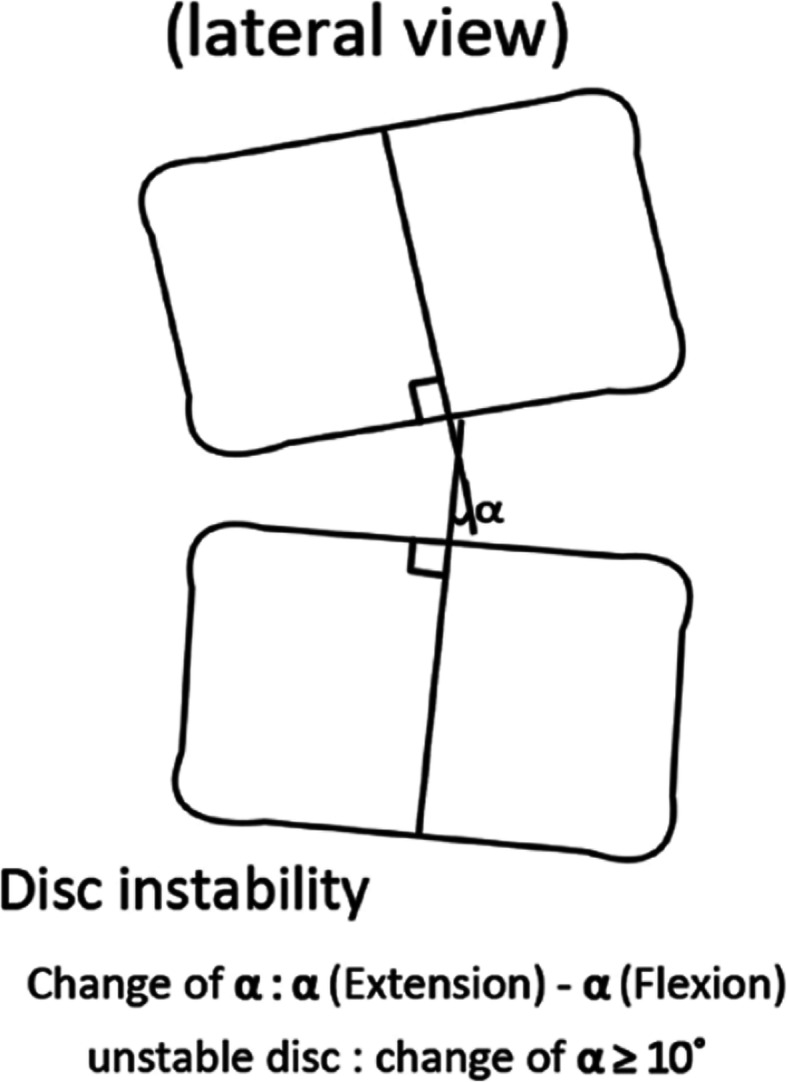
Method for checking for disc instability

All patients were clinically assessed using VAS-B and VAS-L, ranging from no pain (0 point) to worst pain imaginable (10 points). The patients were functionally assessed based on Oswestry disability index (ODI). We compared preoperative and long-term postoperative values. Standing lateral, flexion, and extension radiographs were taken on the patients’ long-term follow-up visit. The Mochida method (Fig. 4) was used to evaluate the disc-height ratio and disc instability [9]. The disc-height ratio and lumbar parameters, such as lumbar lordosis (LL) and sacral slope (SS), at the long-term follow-up visit, were compared to the preoperative value. Based on the study by Sang et al. [8], intervertebral instability was defined as a change > 10° in the angle formed by the superior and inferior disc space of the index level between the flexion and extension radiographs (Fig. 5).

### Statistical analysis

The Statistical Product and Service Solutions (SPSS) 19.0 statistical software (IBM, Armonk, NY, USA) was applied for the statistical analysis, and measurement data were recorded as the mean ± standard deviation (SD). An independent t-test was used to analyze the individual groups, and a chi-square test was used to analyze the enumeration data. A *P*-value < 0.05 was considered statistically significant.

## Results

A total of 267 patients were followed up for at least 3 years after PELD, and 116 patients were followed up after MED. As shown in Table 2, the mean blood loss was significantly lesser in the PELD group (10.8 ± 4.1 mL) compared to that in the MED group (71.3 ± 23.3 mL) (*P* <  0.001), the mean hospital stay was significantly shorter in the PELD group (22.7 ± 4.2 h) compared to that in the MED group (48.1 ± 22.6 h) (*P* <  0.001), and no significant difference was observed in the operating time between the PELD group (78.5 ± 2.6 min) and the MED group (81.1 ± 3.3 min) (*P* = 0.406). The value of VAS-B (0.8 ± 0.4), VAS-L (0.6 ± 0.4), and ODI (12.9 ± 3.2) decreased significantly after PELD than that before the operation at 3 years postoperatively, and the postoperative VAS-B in the PELD group was significantly decreased than that in the MED group (*P* = 0.001).

**Table 2:** Summary of clinical outcomes of the two groups

Observation variables	PELD	MED	***P*** value
**Blood loss (mL)**	10.8 ± 9.0	71.3 ± 23.3	< 0.001
**Operating time (min)**	78.5 ± 32.6	81.1 ± 13.3	0.406
**Hospital stay (hours)**	22.7 ± 4.2	48.1 ± 22.7	< 0.001
**VAS-B**	0.8 ± 0.4	1.1 ± 0.7	0.001
**VAS-L**	0.6 ± 0.4	0.8 ± 0.6	0.224
**ODI (%)**	12.9 ± 3.2	15.1 ± 10.3	0.220
**Complications rate (%)**	9.4	12.1	0.471
**Overall recurrence rate (%)**	6.7	6.0	0.201
**One-year recurrence rate (%)**	5.2	0.9	0.042

*PELD* Percutaneous endoscopic lumbar discectomy, *MED* Microendoscopic discectomy, *VAS-B* Visual analogue scale for the back, *VAS-L* Visual analogue scale for the legs, *ODI* Oswestry dysfunction index

The complications rate was 9.4% (25/267) in the PELD group and 12.1% (14/116) in the MED group, without significant difference (*P* = 0.471). The complications after PELD were detected in 25 cases: 18 cases with recurrence, 5 cases with neural injury, 1 case with dural tear, 1 case with superficial incision infection, and 1 case with wound hematoma. On the other hand, the complications after MED occurred in 14 cases: 7 cases with recurrence, 3 cases with neural injury, 2 cases with dural tear, 1 case with incision infection, and 1 case with wound hematoma. Although no significant difference was observed in the overall postoperative recurrence rate between the two groups, the 1-year postoperative recurrence rate in the PELD group (5.2%, 14/267) was much higher than that in the MED group (0.9%, 1/116) (*P* = 0.042).

As shown in Table 3, the postoperative LL (34.0 ± 10.3) and SS (27.5 ± 5.6) in the PELD group improved significantly compared to that in the MED group (26.9 ± 9.8, 23.6 ± 6.8, respectively; all *P* <  0.001). The disc-height ratio at 3-year follow-up was 85.7 ± 6.4% of the preoperative disc height in the PELD group and 81.9 ± 7.0% in the MED group, with significant height loss in the MED group (*P* = 0.014). Moreover, no intervertebral instability was noted in either of the two groups at the 3-year postoperative follow-up.

**Table 3:** Comparison of radiological outcomes of the two groups

Observation variables	PELD	MED	***P*** value
**Lumbar lordosis (°)**	34.0 ± 10.3	26.9 ± 9.8	< 0.001
**Sacral slope (°)**	27.5 ± 5.6	23.6 ± 6.8	< 0.001
**Disc-height ratio(%)**	85.7 ± 6.4	81.9 ± 7.0	0.014

*PELD* Percutaneous endoscopic lumbar discectomy, *MED* Microendoscopic discectomy

## Discussion

Day surgery, also known as ambulatory surgery, same-day surgery, and one-day surgery, has been carried out in advanced countries in Europe and USA for more than 10 years. Day surgery has advantages of reducing the length of hospital stay, shortening the waiting time for surgery, reducing the length of hospital stay, and reducing the economic burden. PELD has become a popular operative procedure for LDH. The clinical outcome has been reported for each operative approach (interlaminar, transforaminal, and posterolateral) [6, 10, 11]. Although previous studies have shown satisfactory clinical results of PELD in hospitalized patients with LDH, and only a few studies reported about the day surgery patients undergoing PELD.

In this study, we compared the clinical effects between 267 patients treated with PELD in the Day Surgery Unit and contemporary hospitalized 116 patients with LDH treated with MED, all cases were followed up for at least 3 years. The VAS-L, ODI, and complication rates did not differ significantly, which showed that the two kinds of minimally invasive surgery are safe and effective. According to the Mochida method, the disc-height ratio at 3-year follow-up was 85.7% of the preoperative disc height in the PELD group while 81.9% in MED group, which shows that minimally invasive surgery could not avoid disc degeneration and height loss. This phenomenon is consistent with that reported by Sang et al. and Wang et al. [8, 12]. Lumbar instability is a common complication after open discectomy, and the incidence rate is 22%, which could lead to chronic lower back pain [8]. In the current study, although there was no intervertebral instability in both groups after 3-year postoperative follow-ups, the postoperative LL and slopes values in the PELD group improved significantly compared to the values in the MED group. Specifically, although the VAS-B was very low at 3 years postoperatively, the VAS-B was higher significantly in the MED group than that in the PELD group. These findings could be ascribed to the following factors: the paravertebral muscles were stripped, partial vertebral lamina and ligamentum flavum, and the nucleus pulposus was removed in the MED operation with a large working channel.

According to previous literature, the revision rates for PELD were 0.8–9.6% [8, 13, 14], and the revision rates for the MED range was 3.5–10.8% [15]. At the end of 3 years follow-up, the current study identified the revision rate of 6.7% in patients who initially underwent PELD during day surgery. Specifically, the 1-year postoperative recurrence rate in PELD group was 5.2%, which was much higher than that in the MED group (0.9%). Reportedly, the early recurrence (≤ 6 months) rates have been shown to be > 50% in PELD [16]. Surgically unappreciated disc fragment remnants and incomplete decompression by piecemeal removal might lead to a higher early recurrence [13]. The study by Kim et al. showed that total and late recurrence of disc herniation after PEID is associated with advanced age, and the annular sealing technique after fragmentectomy resulted in a lower early recurrence rate compared to PEID without annular sealing [4]. To reduce the recurrence rates, complete removal of the herniated mass is required, including the basal and extruded parts [17].

Nevertheless, the present study has some limitations. First, the data collection was retrospective. Second, the sample size was small. Thus, further prospective randomized controlled clinical trials and long-term follow-up results are essential.

## Conclusions

Although the 1-year postoperative recurrence rate was 5.2%, day surgery for LDH undergoing PELD has several advantages such as less operative blood loss, shorter hospital stay, efficacy for back pain, and effective maintenance of lumbar physiological curvature compared to MED in the long-term follow-up. Therefore, PELD should be considered as valuable day surgery for the treatment of lumbar disc herniation.

## Data Availability

The data and materials in current paper may be made available upon request through sending e-mail to first author.

## Abbreviations

BMI: Body Mass Index
LDH: Lumbar disc herniation
LL: Lumbar lordosis
MED: Microendoscopic discectomy
ODI: Oswestry dysfunction index
PELD: Percutaneous endoscopic lumbar discectomy
SS: Sacral slope
VAS: Visual analogue scale
VAS-B: Visual analogue scale for the back
VAS-L: Visual analogue scale for the legs
